# Synthesis, crystal structure and Hirshfeld surface analysis of a 1:1 salt of sparfloxacin and 4-amino­salicylic acid

**DOI:** 10.1107/S2056989026005736

**Published:** 2026-06-09

**Authors:** Bhumi C. Patel, Krunal M. Modi, J. Prakasha Reddy

**Affiliations:** a Department of Chemistry, School of Sciences, Indrashil University, Rajpur, 382740, India; b Department of Chemisry, School of Engineering, Indrashil University, Rajpur 382740, India; c Department of Chemistry, Faculty of Science, Gokul Global University,Sidhpur, Gujarat, 384151, India; d Department of Applied Chemistry School of Applied Material Sciences Central University of Gujarat, Kundhela 391107 India; Katholieke Universiteit Leuven, Belgium

**Keywords:** crystal structure, sparfloxacinium 4-amino­salicylate salt, Hirshfeld, X-ray diffraction, hydrogen-bonding inter­actions

## Abstract

The anhydrous salt of sparfloxacin [5-amino-1-cyclo­propyl-7-(3,5-dimethylpiperazin-1-yl)-6,8-di­fluoro-4-oxo-1,4-di­hydro­quinoline-3-carb­oxy­lic acid] with 4-amino­salicylic acid, C_22_H_22_F_2_N_4_O_3_^+^·C_7_H_6_NO_3_^−^, features both inter­molecular (N—H⋯O) and intra­molecular (O—H⋯O) inter­actions. In the crystal, two sparfloxacin and two 4-amino­salicylic acid mol­ecules inter­act with each other through N—H⋯O hydrogen bonds, forming an *R*^4^_4_(12) ring motif. The network of inter­molecular inter­actions was further examined using Hirshfeld surface analysis and two-dimensional fingerprint plots.

## Abstract 2

In the crystal structure of the sparfloxacinium 4-amino­salicylate salt, two sparfloxacin and two 4-amino­salicylic acid mol­ecules inter­act with each other through N—H⋯O hydrogen bonds, forming an *R*^4^_4_(12) ring motif.

## 1. Chemical context

Small mol­ecules to peptides are well known for their esthetic appeal (Mehmood *et al.*, 2021 ▸; Patel *et al.*, 2021 ▸) and find various applications including in pharmaceutical chemistry (Shah *et al.*, 2023 ▸; Karmakar *et al.*, 2025 ▸; Gellman, 1998 ▸; Chauhan *et al.*, 2025 ▸). Fluoro­quinones constitute broad spectrum anti­biotics having many advantageous pharmacokinetic properties such as good oral bioavailability and large volume of distribution and are effective against Gram-positive and Gram-negative bacteria (Marona *et al.*, 2001 ▸; Jain *et al.*, 2002 ▸; Faria *et al.*, 2006 ▸). Apart from their use to cure infections in humans, they are also used in veterinary medicine as well as animal husbandry (poultry). A critical review of fluoro­quinones with a focus on respiratory infections was reported (Zhanel *et al.*, 2002 ▸). Sparfloxacin, systematic name: 5-amino-1-cyclo­propyl-7-(3,5-dimethylpiperazin-1-yl)-6,8-di­fluoro-4-oxo-1,4-di­hydro­quinoline-3-carb­oxy­lic acid, C_19_H_22_F_2_N_4_O_3_, is a third-generation fluoro­quinolone anti­biotic, which is one of the most important and successful classes of man-made anti-bacterials with activity against a broad range of bacterial infections especially those affecting the acute exacerbations of chronic bronchitis, urinary tracts, soft tissue infections. bacterial conjunctivitis, *etc*., and prevents bacterial growth primarily by inhibiting the action of DNA gyrase. Reviews of sparfloxacin (Schentag, 2000 ▸), its anti­bacterial activity, pharmacokinetic properties, clinical efficacy, and tolerability in lower respiratory tract infections (Goa *et al.*, 1997 ▸), as well as a review on its penetration into the lower respiratory tract and sinuses have been published (Wise & Honeybourne, 1996 ▸). The electrostatic properties of nine fluoro­quinolone anti­biotics derived directly from their crystal-structure refinements was outlined (Holstein *et al.*, 2012 ▸). Photocatalytic degradation of sparfloxacin using nanoparticles of Ag–TiO_2_ was reported (Kulkarni *et al.*, 2018 ▸). A new validated UV spectrophotometric method for the determination of sparfloxacin in tablets has been described (Sowjanya *et al.*, 2020 ▸). Details of sparfloxacin with inorganic ions CuBr_4_^−^ (Vasil’ev & Golovnev, 2014 ▸), ZnBr_4_^2−^ and CdBr_4_^2−^ (Vasil’ev & Golovnev, 2015 ▸) and BF_4_^−^ (Shingnapurkar *et al.*, 2007 ▸) have also been published. Cocrystals of sparfloxacin with methyl, ethyl, propyl, and isobutyl *para*-hy­droxy­benzoic acids have been reported (Gunnam *et al.*, 2016 ▸). A sparfloxacin salt with pyrocatechuic acid (Zhang *et al.*, 2022 ▸) as well as salts with 2-(carb­oxy­meth­yl)-2-hy­droxy­butane­dioate, pyridine-3-carb­oxyl­ate, 3-carb­oxy­benzoate, 3-carb­oxy­prop-2-enoate, and 2-amino­benzoate anions have been reported (Djaló *et al.*, 2021 ▸). Recently, three salts of sparfloxacin with one of the salts showing extended tapes of fused penta­gonal water assemblies observed were reported (Shankara Prasad *et al.*, 2022 ▸). Continuing our research in the area of cocrystal chemistry (PrakashaReddy & Pedireddi, 2004 ▸), we have synthesized the title compound, which might be a potential solid dosage form if increases in solubility and/or dissolution enhancement are observed.
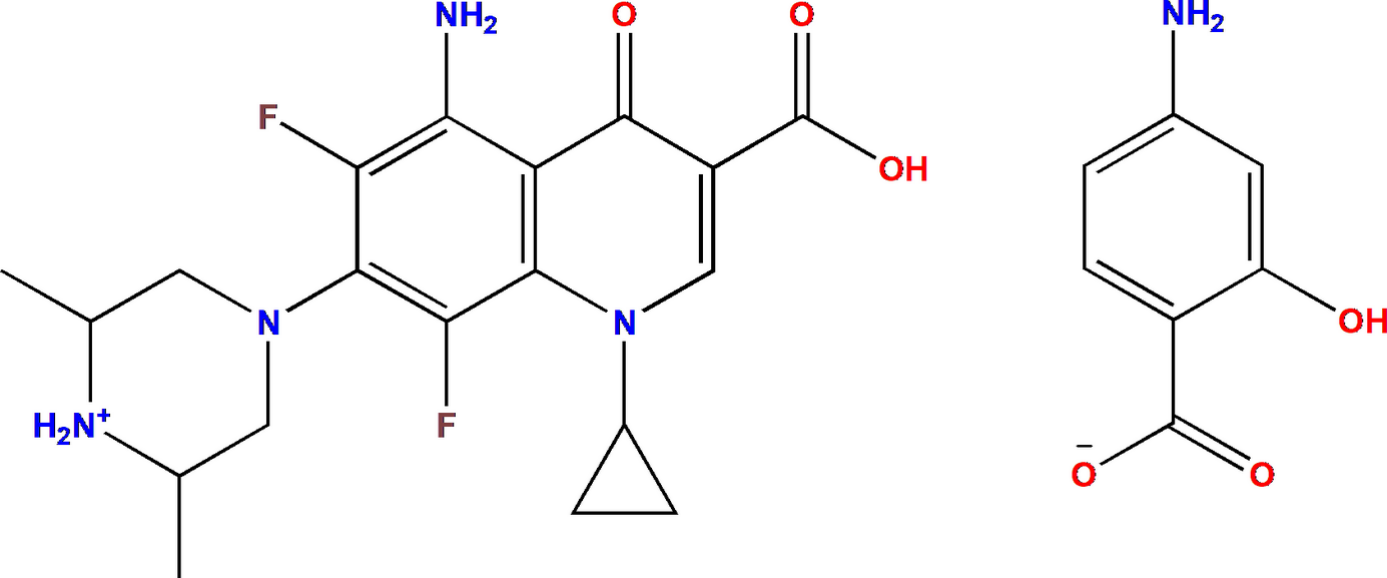


## 2. Structural commentary

**Figure 1:**
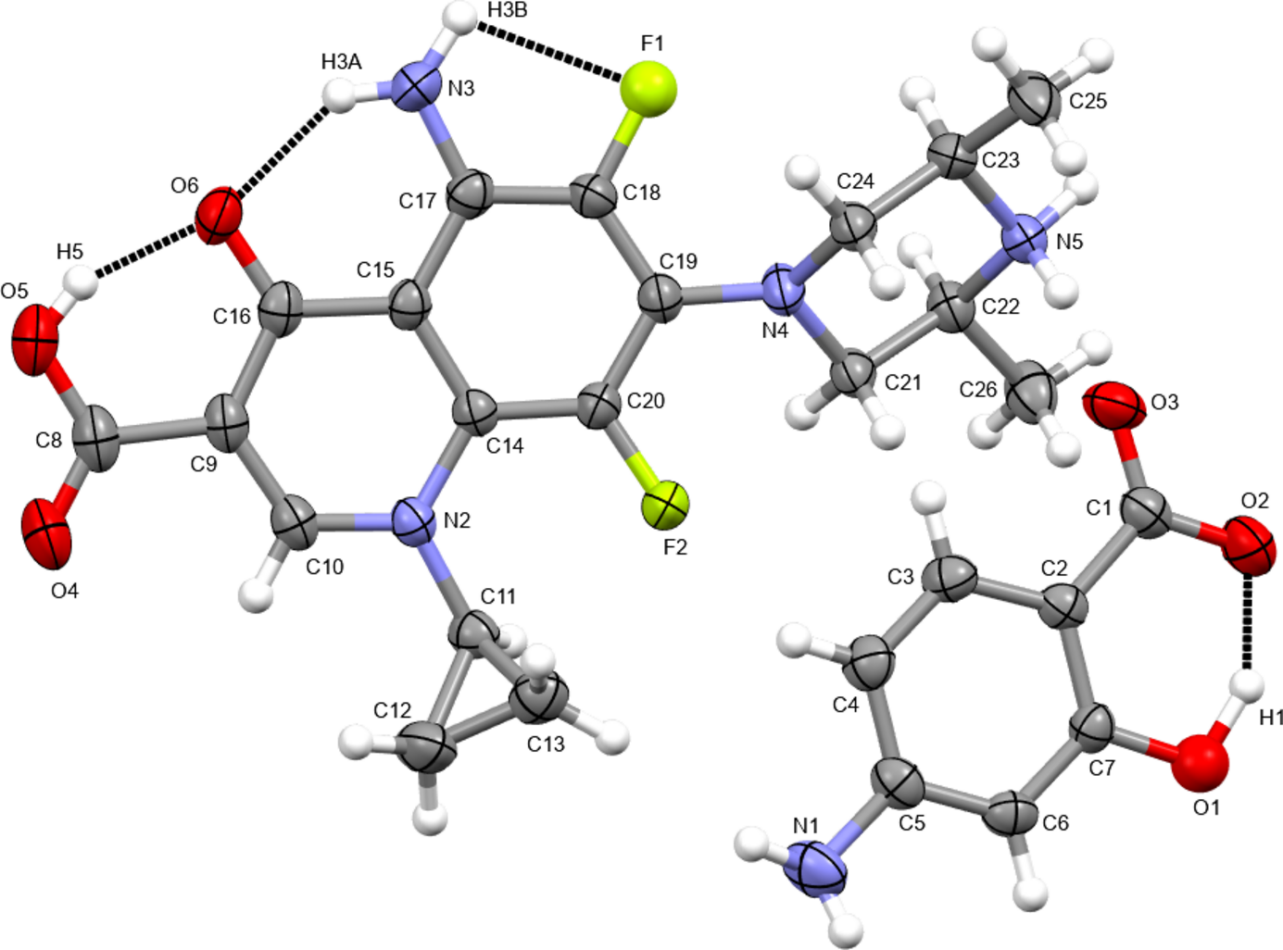
The mol­ecular structure of the sparfloxacinium:4-amino­salicylate salt, showing the atom labelling and displacement ellipsoids drawn at the 30% probability level.

Reaction between sparfloxacin and 4-amino­salicylic acid yielded the title salt, which crystallizes in the monoclinic *P*2_1_/*n* space group with one of each ion in the asymmetric unit. The crystals are solvent free and the mol­ecular structure of the salt along with the atom labelling is shown in Fig. 1 ▸. Structurally, the sparfloxacinium cation is similar to those reported in the literature for other sparfloxacin structures and no unusual bond lengths or angles are observed. The quinoline ring along with the attached carboxyl, amino and fluorine atoms in the sparfloxacinium ion are essentially planar, with an r.m.s. deviation of 0.0631 Å and a largest deviation of 0.1621 (6) Å for atom F1. The dimethyl piperazine ring is oriented away from the quinoline ring, as illustrated by the C18—C19—N4—C24 torsion angle of 54.3 (3)^o^ and the dihedral angle between the best planes through the quinoline ring system and the piperazine ring of 45.61 (9)^o^. For the cyclo­propyl substituent, the C13—C11—N2—C14 torsion angle is 81.1 (3)^o^ and the dihedral angle between the best planes through the quinoline ring system and the cyclo­propyl ring is 54.2 (2)^o^.

The formation of a total of four intra­molecular hydrogen bonds (Table 1 ▸; O1—H1⋯O2, O5—H5⋯O6, N3—H3*B*⋯F1 and N3—H3*A*⋯O6) is observed, formed between the hy­droxy O atom of the –COOH group, the quinoline oxygen atom and the amino group present in sparfloxacin and the hy­droxy group and adjacent oxygen atom present in the 4-amino­salicylic acid, resulting in *S*(5) and *S*(6) ring motifs (Fig. 1 ▸). This formation of intra­molecular ring motifs is preserved, as can also be seen in other salts/co-crystals of sparfloxacin reported in the literature.

## 3. Supra­molecular features

**Figure 2:**
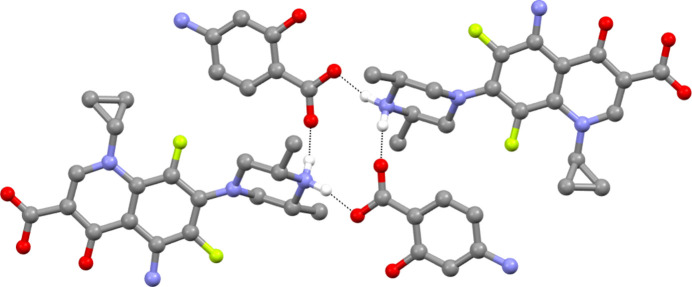
Recognition between the sparfloxacinium:4-amino­salicylate salt through N—H^+^⋯O^−^ inter­actions in the crystal.

**Figure 3:**
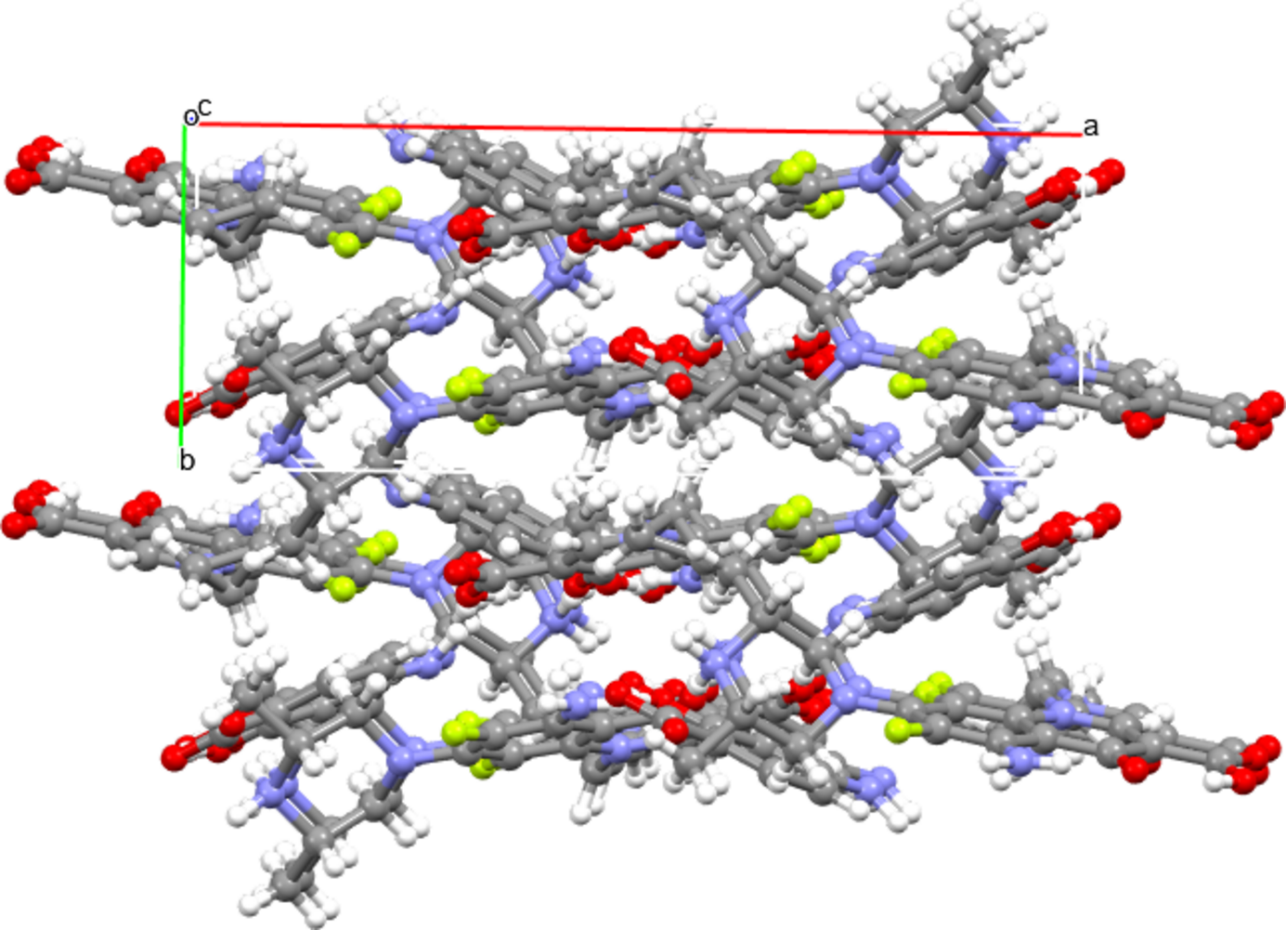
Three-dimensional packing viewed along the *c*-axis direction.

In the crystal structure, a dense network of strong intra- and inter­molecular hydrogen bonding is observed. Crystal-structure analysis revealed that the cation–anion pair recognise through an N—H⋯O hydrogen-bonded 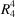
(12) ring motif (Fig. 2 ▸, Table 1 ▸) with their inversion-related counterparts formed between piperazine the NH_2_ group of the cation and the carboxyl­ate group of the anion. These 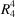
(12) ring motifs are further connected through C—H⋯O hydrogen bonding (Desiraju & Steiner, 1999 ▸; Patel *et al.*, 2024 ▸; Ramesh *et al.*, 2011 ▸). The crystal structure is further consolidated by N—H⋯π (Table 1 ▸) and C=O⋯π [C16=O16⋯*Cg*2; O16⋯*Cg*2 = 3.523 (2) Å; *Cg*2 is the centroid of the pyridine ring N2/C9/C10/C14–C16] inter­actions. In addition, some π–π inter­actions are present in the crystal packing, *e.g*. between pyridine rings with a centroid-to-centroid distance of 3.6378 (12) Å. A three-dimensional projection along the crystallographic *c*-axis direction is shown in Fig. 3 ▸.

## 4. Hirshfeld surfaces and two-dimensional fingerprint plots

**Figure 4:**
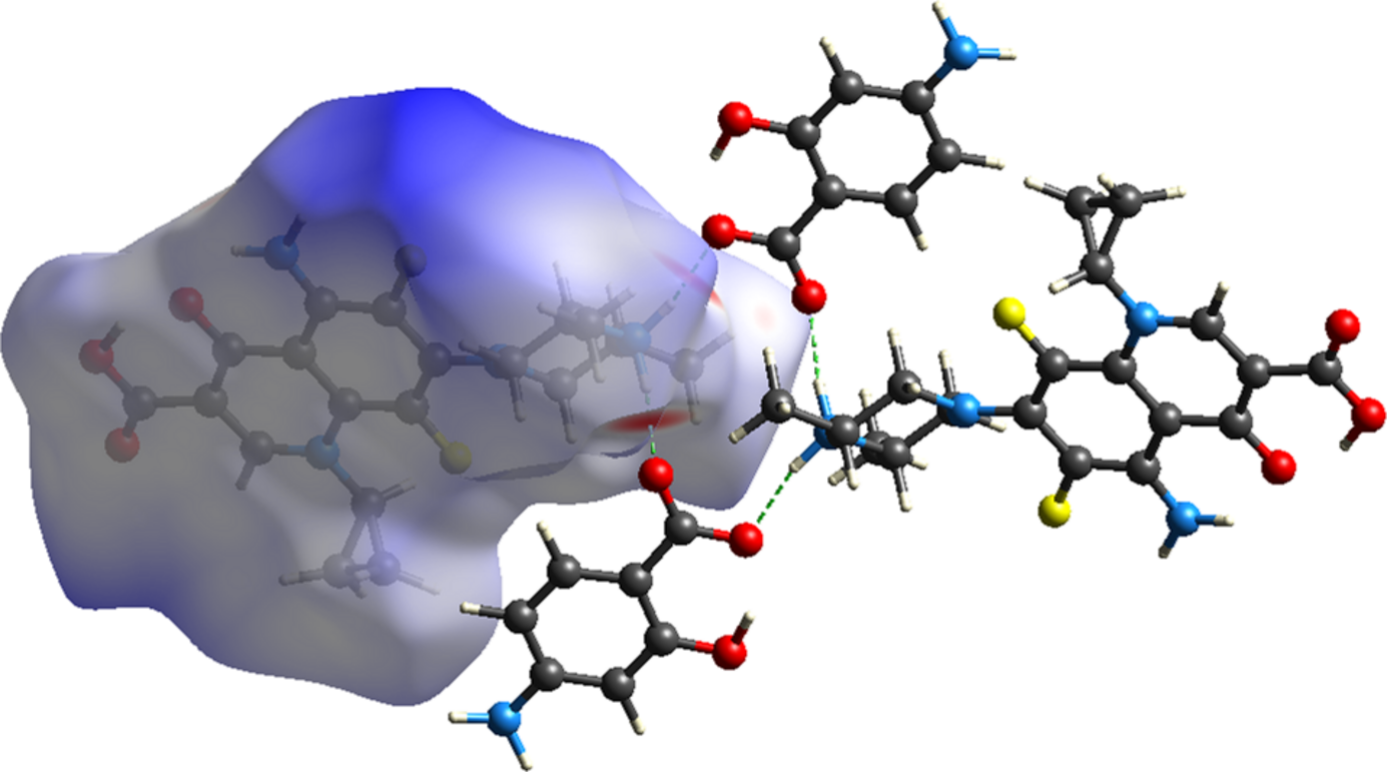
Hirshfeld surface mapped over *d*_norm_ showing N—H^+^⋯O^−^inter­molecular contacts.

**Figure 5:**
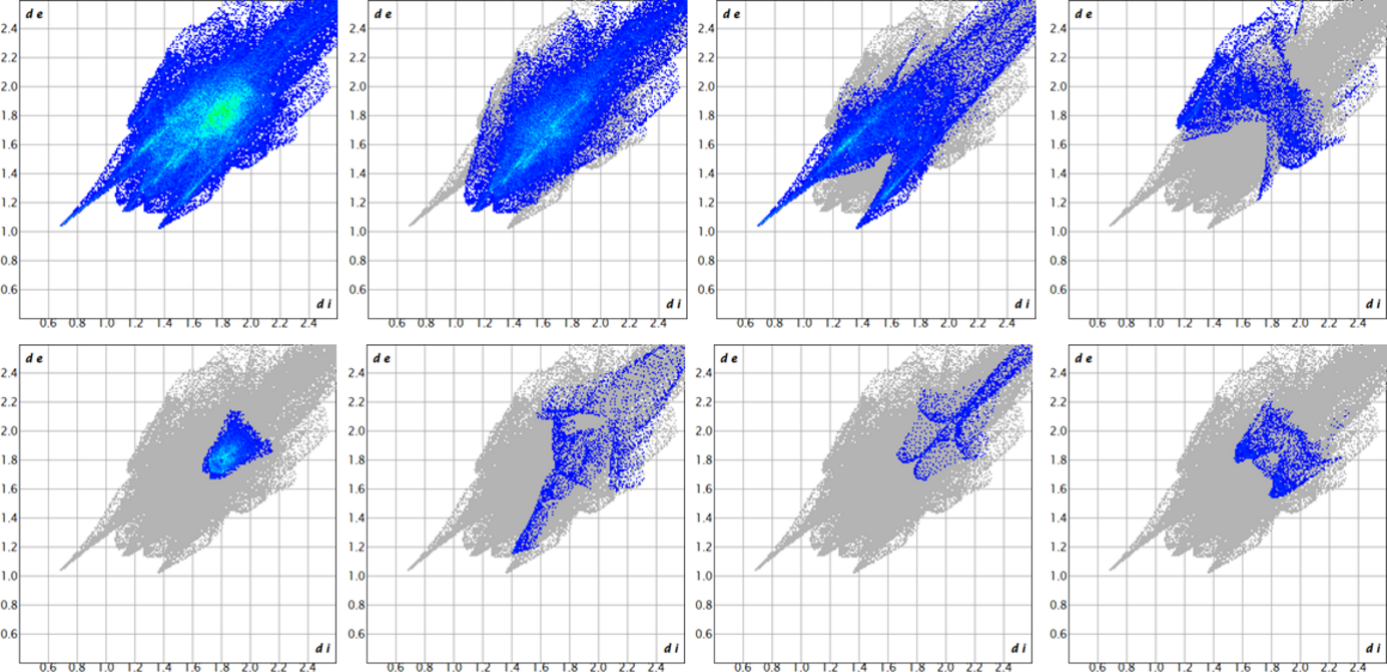
The full two-dimensional fingerprint plot for the title salt and those delineated into H⋯H (46.3%), O⋯H/H⋯O (25.7%), C⋯H/H⋯C (7.1%), C⋯C (6.7%), F⋯H/H⋯F (5.4%), F⋯O/O⋯F (2.8%) and C⋯O/O⋯C (2.7%) contacts.

A Hirshfeld surface analysis and the corresponding fingerprint plots were generated using *CrystalExplorer* software (Spackman *et al.*, 2021 ▸; Spackman & Jayatilaka, 2009 ▸) to further investigate and qu­antify the contributions of the various inter­molecular inter­actions in the crystal. The Hirshfeld surface mapped over *d*_norm_ and corresponding colours representing various inter­actions are shown in Fig. 4 ▸. The two-dimensional fingerprint plots (McKinnon *et al.*, 2007 ▸) for all inter­molecular inter­actions and those delineated into specific contacts are shown in Fig. 5 ▸. The largest contribution comes from H⋯H contacts at 46.3% of the total, which is consistent with the significant hydrogen content of the mol­ecule. The next most important contact is O⋯H/H⋯O at 25.7%, which primarily comes from the intra­molecular O—H⋯O and inter­molecular N—H⋯O as well as C—H⋯O inter­actions. The C⋯H/H⋯C inter­actions account for 7.1% while C⋯C contacts contribute 6.7%, followed by F⋯H/H⋯F contacts contributing 5.4%. Further, 2.8 and 2.7% contributions corresponding to F⋯O/O⋯F and C⋯O/O⋯C contacts, respectively, are also observed.

## 5. Synthesis and crystallization

Sparfloxacin and 4-amino­salicylic acid were obtained from Aldrich, and HPLC-grade methanol was used for reaction. Sparfloxacin (100 mg, 0.255 mmol) was dissolved in methanol (10 ml) under constant stirring at 330 K for 30 min. An equimolar solution of 4-amino­salicylic acid (39 mg, 0.255 mmol) in methanol (10 ml) was added to the solution of sparfloxacin and stirring was continued further for about 30 min at 330 K. The mixture was cooled to room temperature and the solution was filtered. X-ray quality single crystals of suitable dimension were obtained over a period of five days by slow evaporation of the solvent.

## 6. Refinement

Crystal data, data collection and structure refinement details are summarized in Table 2 ▸. All hydrogen atoms were placed at idealized positions and refined using a riding model.

## Supplementary Material

Crystal structure: contains datablock(s) I. DOI: 10.1107/S2056989026005736/vm2330sup1.cif

Structure factors: contains datablock(s) I. DOI: 10.1107/S2056989026005736/vm2330Isup2.hkl

CCDC reference: 2558235

Additional supporting information:  crystallographic information; 3D view; checkCIF report

## Figures and Tables

**Table 1:** Hydrogen-bond geometry (Å, °) *Cg*6 is the centroid of the C2–C7 ring.

*D*—H⋯*A*	*D*—H	H⋯*A*	*D*⋯*A*	*D*—H⋯*A*
O1—H1⋯O2	1.02 (4)	1.60 (4)	2.529 (3)	149 (3)
N3—H3*A*⋯O6	0.86	1.92	2.648 (3)	142
N3—H3*A*⋯O1^i^	0.86	2.52	2.965 (3)	113
N3—H3*B*⋯F1	0.91 (3)	2.29 (3)	2.637 (3)	102 (2)
O5—H5⋯O6	0.82	1.73	2.497 (3)	154
N5—H5*A*⋯O2^ii^	0.99 (3)	1.74 (3)	2.715 (3)	167 (2)
N5—H5*B*⋯O3	0.91 (3)	1.85 (3)	2.754 (3)	172 (3)
N1—H1*B*⋯*Cg*6^iii^	0.95 (4)	2.46 (5)	3.326 (3)	153 (4)

Symmetry codes: (i) 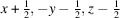; (ii) 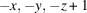; (iii) 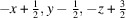.

**Table 2:** Experimental details

Crystal data	
Chemical formula	C_19_H_23_F_2_N_4_O_3_^+^·C_7_H_6_NO_3_^−^
*M*_r_	545.54
Crystal system, space group	Monoclinic, *P*2_1_/*n*
Temperature (K)	296
*a*, *b*, *c* (Å)	18.7603 (3), 7.15744 (10), 20.8511 (3)
β (°)	94.3739 (14)
*V* (Å^3^)	2791.65 (7)
*Z*	4
Radiation type	Cu *K*α
μ (mm^−1^)	0.87
Crystal size (mm)	0.29 × 0.21 × 0.12

Data collection	
Diffractometer	XtaLAB Synergy, Dualflex, HyPix
Absorption correction	Multi-scan (*CrysAlis PRO*; Rigaku OD, 2024 ▸)
*T*_min_, *T*_max_	0.416, 1.000
No. of measured, independent and observed [*I* > 2σ(*I*)] reflections	26903, 5468, 4535
*R*_int_	0.030
(sin θ/λ)_max_ (Å^−1^)	0.624

Refinement	
*R*[*F*^2^ > 2σ(*F*^2^)], *wR*(*F*^2^), *S*	0.064, 0.199, 1.08
No. of reflections	5468
No. of parameters	368
H-atom treatment	H atoms treated by a mixture of independent and constrained refinement
Δρ_max_, Δρ_min_ (e Å^−3^)	0.86, −0.72

Computer programs: *CrysAlis PRO* (Rigaku OD, 2024 ▸), *OLEX2.solve* (Bourhis *et al.*, 2015 ▸), *SHELXL2019/3* (Sheldrick, 2015 ▸) and *OLEX2* (Dolomanov *et al.*, 2009 ▸).

## supplementary crystallographic information

### 4-[5-Amino-3-carboxy-1-cyclopropyl-6,8-difluoro-4-oxo-1,4-dihydroquinolin-7-yl]-2,6-dimethylpiperazin-1-ium 4-amino-2-hydroxybenzoate Crystal data

**Table:**

C_19_H_23_F_2_N_4_O_3_^+^·C_7_H_6_NO_3_^−^	*F*(000) = 1144
*M**_r_* = 545.54	*D*_x_ = 1.298 Mg m^−^^3^
Monoclinic, *P*2_1_/*n*	Cu *K*α radiation, λ = 1.54184 Å
*a* = 18.7603 (3) Å	Cell parameters from 15825 reflections
*b* = 7.15744 (10) Å	θ = 3.0–74.2°
*c* = 20.8511 (3) Å	µ = 0.87 mm^−^^1^
β = 94.3739 (14)°	*T* = 296 K
*V* = 2791.65 (7) Å^3^	Block, colourless
*Z* = 4	0.29 × 0.21 × 0.12 mm

### 4-[5-Amino-3-carboxy-1-cyclopropyl-6,8-difluoro-4-oxo-1,4-dihydroquinolin-7-yl]-2,6-dimethylpiperazin-1-ium 4-amino-2-hydroxybenzoate Data collection

**Table:**

XtaLAB Synergy, Dualflex, HyPix diffractometer	4535 reflections with *I* > 2σ(*I*)
Detector resolution: 10.0000 pixels mm^-1^	*R*_int_ = 0.030
ω scans	θ_max_ = 74.3°, θ_min_ = 3.1°
Absorption correction: multi-scan (CrysAlisPro; Rigaku OD, 2024)	*h* = −22→23
*T*_min_ = 0.416, *T*_max_ = 1.000	*k* = −6→8
26903 measured reflections	*l* = −26→25
5468 independent reflections	

### 4-[5-Amino-3-carboxy-1-cyclopropyl-6,8-difluoro-4-oxo-1,4-dihydroquinolin-7-yl]-2,6-dimethylpiperazin-1-ium 4-amino-2-hydroxybenzoate Refinement

**Table:**

Refinement on *F*^2^	Hydrogen site location: mixed
Least-squares matrix: full	H atoms treated by a mixture of independent and constrained refinement
*R*[*F*^2^ > 2σ(*F*^2^)] = 0.064	*w* = 1/[σ^2^(*F*_o_^2^) + (0.1004*P*)^2^ + 1.2039*P*] where *P* = (*F*_o_^2^ + 2*F*_c_^2^)/3
*wR*(*F*^2^) = 0.199	(Δ/σ)_max_ = 0.001
*S* = 1.08	Δρ_max_ = 0.86 e Å^−^^3^
5468 reflections	Δρ_min_ = −0.72 e Å^−^^3^
368 parameters	Extinction correction: SHELXL2019/2 (Sheldrick 2015), Fc^*^=kFc[1+0.001xFc^2^λ^3^/sin(2θ)]^-1/4^
0 restraints	Extinction coefficient: 0.00063 (19)

### 4-[5-Amino-3-carboxy-1-cyclopropyl-6,8-difluoro-4-oxo-1,4-dihydroquinolin-7-yl]-2,6-dimethylpiperazin-1-ium 4-amino-2-hydroxybenzoate Special details

**Table:**

Geometry. All esds (except the esd in the dihedral angle between two l.s. planes) are estimated using the full covariance matrix. The cell esds are taken into account individually in the estimation of esds in distances, angles and torsion angles; correlations between esds in cell parameters are only used when they are defined by crystal symmetry. An approximate (isotropic) treatment of cell esds is used for estimating esds involving l.s. planes.

### 4-[5-Amino-3-carboxy-1-cyclopropyl-6,8-difluoro-4-oxo-1,4-dihydroquinolin-7-yl]-2,6-dimethylpiperazin-1-ium 4-amino-2-hydroxybenzoate Fractional atomic coordinates and isotropic or equivalent isotropic displacement parameters (Å^2^)

**Table:**

	*x*	*y*	*z*	*U*_iso_*/*U*_eq_	
F2	0.33141 (7)	−0.1307 (3)	0.58770 (6)	0.0743 (4)	
O6	0.56631 (9)	−0.3390 (3)	0.42044 (9)	0.0680 (5)	
N2	0.48134 (9)	−0.1866 (3)	0.58847 (9)	0.0507 (4)	
O5	0.68550 (9)	−0.3691 (3)	0.48149 (11)	0.0796 (6)	
H5	0.653515	−0.370703	0.452402	0.119*	
O2	−0.01246 (11)	−0.1420 (3)	0.63440 (10)	0.0835 (6)	
N5	0.09720 (10)	−0.0477 (3)	0.45468 (10)	0.0546 (4)	
H5A	0.0611 (14)	0.020 (4)	0.4266 (13)	0.066*	
H5B	0.0779 (14)	−0.110 (4)	0.4874 (14)	0.066*	
C20	0.36168 (11)	−0.1669 (3)	0.53233 (10)	0.0513 (5)	
C14	0.43458 (11)	−0.1997 (3)	0.53226 (10)	0.0465 (5)	
O3	0.05206 (11)	−0.2463 (3)	0.55722 (9)	0.0800 (6)	
O4	0.69731 (10)	−0.3034 (3)	0.58457 (12)	0.0906 (7)	
N4	0.24237 (9)	−0.1468 (3)	0.47622 (10)	0.0592 (5)	
C17	0.41696 (12)	−0.2660 (3)	0.41633 (11)	0.0537 (5)	
C24	0.19089 (12)	−0.2854 (3)	0.45037 (12)	0.0563 (5)	
H24A	0.173551	−0.357309	0.485410	0.068*	
H24B	0.214234	−0.370730	0.422507	0.068*	
C16	0.53884 (12)	−0.2912 (3)	0.47192 (12)	0.0533 (5)	
N3	0.43841 (13)	−0.3174 (4)	0.35779 (11)	0.0724 (6)	
H3A	0.484477	−0.321564	0.359819	0.087*	
C19	0.31507 (11)	−0.1793 (3)	0.47702 (11)	0.0514 (5)	
C2	0.09910 (12)	−0.2787 (3)	0.66502 (10)	0.0512 (5)	
C9	0.58140 (11)	−0.2758 (3)	0.53079 (12)	0.0555 (5)	
C18	0.34517 (12)	−0.2265 (3)	0.42088 (10)	0.0544 (5)	
C10	0.55099 (12)	−0.2224 (3)	0.58527 (12)	0.0556 (5)	
H10	0.580734	−0.210068	0.622802	0.067*	
C21	0.21160 (11)	−0.0136 (3)	0.51888 (11)	0.0545 (5)	
H21A	0.247947	0.074656	0.534928	0.065*	
H21B	0.194252	−0.078839	0.555368	0.065*	
C15	0.46329 (11)	−0.2517 (3)	0.47310 (10)	0.0487 (5)	
C7	0.09308 (12)	−0.2443 (3)	0.73051 (11)	0.0519 (5)	
C11	0.45681 (12)	−0.1226 (3)	0.64947 (11)	0.0562 (5)	
H11	0.439981	0.006979	0.649617	0.067*	
C5	0.20952 (13)	−0.3765 (3)	0.75775 (12)	0.0588 (6)	
C3	0.16136 (13)	−0.3667 (3)	0.64840 (11)	0.0592 (6)	
H3	0.166266	−0.393645	0.605331	0.071*	
C6	0.14795 (13)	−0.2905 (3)	0.77576 (11)	0.0574 (5)	
H6	0.143489	−0.263401	0.818898	0.069*	
N1	0.26501 (17)	−0.4184 (4)	0.80268 (15)	0.0834 (8)	
C8	0.65916 (13)	−0.3161 (4)	0.53528 (16)	0.0687 (7)	
C23	0.12860 (12)	−0.1927 (4)	0.41281 (11)	0.0579 (5)	
H23	0.146013	−0.130730	0.375111	0.069*	
C4	0.21548 (14)	−0.4149 (3)	0.69294 (12)	0.0636 (6)	
H4	0.256253	−0.473365	0.679945	0.076*	
C1	0.04328 (13)	−0.2201 (4)	0.61521 (12)	0.0608 (6)	
C22	0.15077 (12)	0.0897 (3)	0.48317 (11)	0.0567 (5)	
H22	0.169622	0.162133	0.448340	0.068*	
C26	0.11441 (15)	0.2223 (4)	0.52730 (15)	0.0770 (8)	
H26A	0.074505	0.281075	0.503798	0.116*	
H26B	0.147855	0.316011	0.543166	0.116*	
H26C	0.097888	0.153527	0.562811	0.116*	
C25	0.07087 (15)	−0.3306 (5)	0.39044 (15)	0.0793 (8)	
H25A	0.053401	−0.391960	0.427059	0.119*	
H25B	0.090274	−0.421790	0.362854	0.119*	
H25C	0.032303	−0.265493	0.367218	0.119*	
C12	0.49327 (18)	−0.1882 (5)	0.71117 (14)	0.0880 (9)	
H12A	0.531824	−0.277755	0.709146	0.106*	
H12B	0.499333	−0.099296	0.746292	0.106*	
C13	0.41968 (16)	−0.2517 (4)	0.69119 (13)	0.0728 (7)	
H13A	0.413371	−0.380169	0.677069	0.087*	
H13B	0.380889	−0.201766	0.714203	0.087*	
F1	0.30262 (8)	−0.2317 (2)	0.36541 (7)	0.0718 (4)*	
O1	0.03417 (11)	−0.1655 (3)	0.75118 (10)	0.0753 (5)*	
H1	0.000 (2)	−0.157 (6)	0.7110 (19)	0.113*	
H3B	0.4064 (16)	−0.320 (4)	0.3226 (15)	0.081 (9)*	
H1A	0.252 (2)	−0.421 (6)	0.8376 (18)	0.102 (14)*	
H1B	0.298 (2)	−0.509 (7)	0.790 (2)	0.123 (14)*	

### 4-[5-Amino-3-carboxy-1-cyclopropyl-6,8-difluoro-4-oxo-1,4-dihydroquinolin-7-yl]-2,6-dimethylpiperazin-1-ium 4-amino-2-hydroxybenzoate Atomic displacement parameters (Å^2^)

**Table:**

	*U*^11^	*U*^22^	*U*^33^	*U*^12^	*U*^13^	*U*^23^
F2	0.0503 (7)	0.1215 (13)	0.0515 (7)	0.0121 (8)	0.0064 (6)	−0.0052 (8)
O6	0.0546 (9)	0.0785 (11)	0.0734 (11)	0.0087 (8)	0.0207 (8)	0.0036 (9)
N2	0.0463 (9)	0.0521 (9)	0.0529 (10)	0.0015 (7)	−0.0011 (7)	0.0038 (8)
O5	0.0489 (9)	0.0809 (12)	0.1107 (15)	0.0062 (9)	0.0169 (10)	0.0025 (12)
O2	0.0698 (12)	0.0990 (14)	0.0793 (12)	0.0197 (11)	−0.0107 (10)	0.0046 (11)
N5	0.0446 (9)	0.0653 (11)	0.0531 (10)	0.0042 (8)	−0.0021 (8)	−0.0006 (9)
C20	0.0456 (11)	0.0588 (12)	0.0501 (11)	0.0018 (9)	0.0081 (9)	0.0010 (9)
C14	0.0442 (10)	0.0448 (10)	0.0503 (11)	−0.0007 (8)	0.0020 (8)	0.0060 (8)
O3	0.0837 (13)	0.1012 (15)	0.0528 (10)	−0.0111 (11)	−0.0094 (9)	0.0077 (9)
O4	0.0486 (10)	0.1061 (16)	0.1147 (17)	0.0052 (10)	−0.0091 (11)	0.0005 (13)
N4	0.0409 (9)	0.0695 (12)	0.0668 (12)	0.0016 (8)	0.0005 (8)	−0.0154 (9)
C17	0.0549 (12)	0.0547 (12)	0.0524 (12)	−0.0011 (9)	0.0096 (10)	0.0057 (9)
C24	0.0461 (11)	0.0635 (13)	0.0587 (12)	0.0001 (10)	0.0010 (9)	−0.0075 (10)
C16	0.0478 (11)	0.0462 (10)	0.0670 (13)	0.0010 (9)	0.0107 (10)	0.0088 (9)
N3	0.0593 (12)	0.1066 (18)	0.0525 (11)	0.0027 (12)	0.0129 (10)	−0.0044 (11)
C19	0.0431 (10)	0.0543 (11)	0.0565 (12)	0.0005 (9)	0.0027 (9)	0.0039 (9)
C2	0.0538 (12)	0.0488 (11)	0.0504 (11)	−0.0043 (9)	0.0006 (9)	0.0044 (9)
C9	0.0419 (11)	0.0501 (11)	0.0745 (15)	0.0003 (9)	0.0044 (10)	0.0086 (10)
C18	0.0508 (12)	0.0657 (13)	0.0459 (11)	−0.0004 (10)	−0.0021 (9)	0.0038 (9)
C10	0.0470 (11)	0.0520 (11)	0.0666 (14)	−0.0004 (9)	−0.0034 (10)	0.0062 (10)
C21	0.0432 (10)	0.0605 (12)	0.0590 (12)	−0.0011 (9)	−0.0012 (9)	−0.0072 (10)
C15	0.0448 (10)	0.0471 (10)	0.0548 (12)	0.0000 (8)	0.0077 (9)	0.0066 (9)
C7	0.0501 (11)	0.0528 (11)	0.0538 (12)	0.0000 (9)	0.0098 (9)	0.0036 (9)
C11	0.0584 (12)	0.0558 (12)	0.0536 (12)	0.0076 (10)	−0.0010 (10)	−0.0010 (10)
C5	0.0625 (13)	0.0499 (11)	0.0627 (13)	0.0022 (10)	−0.0039 (10)	0.0090 (10)
C3	0.0700 (14)	0.0560 (12)	0.0519 (12)	0.0046 (11)	0.0072 (10)	−0.0024 (10)
C6	0.0678 (14)	0.0590 (12)	0.0456 (11)	−0.0029 (11)	0.0047 (10)	0.0060 (9)
N1	0.0879 (18)	0.0835 (17)	0.0752 (17)	0.0218 (14)	−0.0170 (14)	0.0084 (14)
C8	0.0461 (12)	0.0625 (14)	0.097 (2)	0.0009 (10)	0.0032 (13)	0.0092 (13)
C23	0.0524 (12)	0.0742 (14)	0.0465 (11)	0.0015 (11)	−0.0002 (9)	−0.0048 (10)
C4	0.0650 (14)	0.0589 (13)	0.0673 (14)	0.0125 (11)	0.0070 (11)	0.0006 (11)
C1	0.0585 (13)	0.0636 (13)	0.0587 (13)	−0.0062 (11)	−0.0051 (11)	0.0079 (11)
C22	0.0488 (11)	0.0592 (12)	0.0615 (13)	0.0003 (10)	−0.0001 (10)	−0.0007 (10)
C26	0.0633 (15)	0.0695 (16)	0.096 (2)	0.0115 (12)	−0.0062 (14)	−0.0209 (15)
C25	0.0602 (15)	0.094 (2)	0.0810 (18)	0.0015 (14)	−0.0139 (13)	−0.0276 (15)
C12	0.089 (2)	0.117 (2)	0.0573 (15)	0.0244 (19)	−0.0056 (14)	0.0011 (16)
C13	0.0813 (18)	0.0751 (16)	0.0629 (15)	0.0115 (13)	0.0117 (13)	0.0125 (12)

### 4-[5-Amino-3-carboxy-1-cyclopropyl-6,8-difluoro-4-oxo-1,4-dihydroquinolin-7-yl]-2,6-dimethylpiperazin-1-ium 4-amino-2-hydroxybenzoate Geometric parameters (Å, º)

**Table:**

F2—C20	1.350 (2)	C18—F1	1.355 (3)
O6—C16	1.272 (3)	C10—H10	0.9300
N2—C14	1.413 (3)	C21—H21A	0.9700
N2—C10	1.338 (3)	C21—H21B	0.9700
N2—C11	1.459 (3)	C21—C22	1.508 (3)
O5—H5	0.8200	C7—C6	1.382 (3)
O5—C8	1.315 (4)	C7—O1	1.341 (3)
O2—C1	1.277 (3)	C11—H11	0.9800
N5—H5A	0.99 (3)	C11—C12	1.486 (3)
N5—H5B	0.91 (3)	C11—C13	1.479 (4)
N5—C23	1.505 (3)	C5—C6	1.386 (3)
N5—C22	1.496 (3)	C5—N1	1.379 (3)
C20—C14	1.388 (3)	C5—C4	1.392 (3)
C20—C19	1.396 (3)	C3—H3	0.9300
C14—C15	1.432 (3)	C3—C4	1.367 (3)
O3—C1	1.247 (3)	C6—H6	0.9300
O4—C8	1.210 (4)	N1—H1A	0.78 (4)
N4—C24	1.459 (3)	N1—H1B	0.96 (5)
N4—C19	1.382 (3)	C23—H23	0.9800
N4—C21	1.453 (3)	C23—C25	1.512 (4)
C17—N3	1.365 (3)	C4—H4	0.9300
C17—C18	1.386 (3)	C22—H22	0.9800
C17—C15	1.418 (3)	C22—C26	1.519 (3)
C24—H24A	0.9700	C26—H26A	0.9600
C24—H24B	0.9700	C26—H26B	0.9600
C24—C23	1.510 (3)	C26—H26C	0.9600
C16—C9	1.417 (3)	C25—H25A	0.9600
C16—C15	1.447 (3)	C25—H25B	0.9600
N3—H3A	0.8626	C25—H25C	0.9600
N3—H3B	0.91 (3)	C12—H12A	0.9700
C19—C18	1.380 (3)	C12—H12B	0.9700
C2—C7	1.401 (3)	C12—C13	1.483 (5)
C2—C3	1.394 (3)	C13—H13A	0.9700
C2—C1	1.478 (3)	C13—H13B	0.9700
C9—C10	1.364 (4)	O1—H1	1.02 (4)
C9—C8	1.483 (3)		
			
C14—N2—C11	121.80 (17)	N2—C11—C13	120.7 (2)
C10—N2—C14	119.53 (19)	C12—C11—H11	115.0
C10—N2—C11	118.54 (19)	C13—C11—H11	115.0
C8—O5—H5	109.5	C13—C11—C12	60.0 (2)
H5A—N5—H5B	113 (2)	C6—C5—C4	118.8 (2)
C23—N5—H5A	106.0 (15)	N1—C5—C6	120.9 (3)
C23—N5—H5B	107.1 (17)	N1—C5—C4	120.3 (3)
C22—N5—H5A	108.8 (16)	C2—C3—H3	118.7
C22—N5—H5B	108.4 (17)	C4—C3—C2	122.6 (2)
C22—N5—C23	113.76 (17)	C4—C3—H3	118.7
F2—C20—C14	120.82 (19)	C7—C6—C5	120.9 (2)
F2—C20—C19	116.15 (18)	C7—C6—H6	119.5
C14—C20—C19	122.9 (2)	C5—C6—H6	119.5
N2—C14—C15	118.78 (18)	C5—N1—H1A	112 (3)
C20—C14—N2	122.52 (19)	C5—N1—H1B	116 (2)
C20—C14—C15	118.70 (19)	H1A—N1—H1B	120 (4)
C19—N4—C24	120.98 (19)	O5—C8—C9	115.7 (2)
C19—N4—C21	122.72 (18)	O4—C8—O5	120.6 (2)
C21—N4—C24	112.94 (17)	O4—C8—C9	123.6 (3)
N3—C17—C18	118.1 (2)	N5—C23—C24	108.90 (18)
N3—C17—C15	124.2 (2)	N5—C23—H23	108.8
C18—C17—C15	117.7 (2)	N5—C23—C25	109.0 (2)
N4—C24—H24A	109.5	C24—C23—H23	108.8
N4—C24—H24B	109.5	C24—C23—C25	112.4 (2)
N4—C24—C23	110.9 (2)	C25—C23—H23	108.8
H24A—C24—H24B	108.0	C5—C4—H4	120.0
C23—C24—H24A	109.5	C3—C4—C5	119.9 (2)
C23—C24—H24B	109.5	C3—C4—H4	120.0
O6—C16—C9	120.9 (2)	O2—C1—C2	117.2 (2)
O6—C16—C15	121.8 (2)	O3—C1—O2	122.8 (2)
C9—C16—C15	117.3 (2)	O3—C1—C2	120.0 (2)
C17—N3—H3A	109.2	N5—C22—C21	109.50 (19)
C17—N3—H3B	120.3 (19)	N5—C22—H22	108.8
H3A—N3—H3B	129.4	N5—C22—C26	109.40 (19)
N4—C19—C20	123.7 (2)	C21—C22—H22	108.8
C18—C19—C20	116.44 (19)	C21—C22—C26	111.4 (2)
C18—C19—N4	119.9 (2)	C26—C22—H22	108.8
C7—C2—C1	122.0 (2)	C22—C26—H26A	109.5
C3—C2—C7	117.0 (2)	C22—C26—H26B	109.5
C3—C2—C1	121.0 (2)	C22—C26—H26C	109.5
C16—C9—C8	121.7 (2)	H26A—C26—H26B	109.5
C10—C9—C16	120.0 (2)	H26A—C26—H26C	109.5
C10—C9—C8	118.3 (2)	H26B—C26—H26C	109.5
C19—C18—C17	124.8 (2)	C23—C25—H25A	109.5
F1—C18—C17	116.6 (2)	C23—C25—H25B	109.5
F1—C18—C19	118.6 (2)	C23—C25—H25C	109.5
N2—C10—C9	124.6 (2)	H25A—C25—H25B	109.5
N2—C10—H10	117.7	H25A—C25—H25C	109.5
C9—C10—H10	117.7	H25B—C25—H25C	109.5
N4—C21—H21A	109.7	C11—C12—H12A	117.8
N4—C21—H21B	109.7	C11—C12—H12B	117.8
N4—C21—C22	109.91 (18)	H12A—C12—H12B	114.9
H21A—C21—H21B	108.2	C13—C12—C11	59.76 (18)
C22—C21—H21A	109.7	C13—C12—H12A	117.8
C22—C21—H21B	109.7	C13—C12—H12B	117.8
C14—C15—C16	119.9 (2)	C11—C13—C12	60.24 (19)
C17—C15—C14	119.41 (19)	C11—C13—H13A	117.7
C17—C15—C16	120.7 (2)	C11—C13—H13B	117.7
C6—C7—C2	120.8 (2)	C12—C13—H13A	117.7
O1—C7—C2	121.1 (2)	C12—C13—H13B	117.7
O1—C7—C6	118.1 (2)	H13A—C13—H13B	114.9
N2—C11—H11	115.0	C7—O1—H1	104 (2)
N2—C11—C12	120.0 (2)		
			
F2—C20—C14—N2	−3.8 (3)	C2—C3—C4—C5	0.1 (4)
F2—C20—C14—C15	175.22 (19)	C9—C16—C15—C14	−0.1 (3)
F2—C20—C19—N4	3.7 (3)	C9—C16—C15—C17	−179.30 (19)
F2—C20—C19—C18	−176.5 (2)	C18—C17—C15—C14	0.6 (3)
O6—C16—C9—C10	179.3 (2)	C18—C17—C15—C16	179.9 (2)
O6—C16—C9—C8	−0.6 (3)	C10—N2—C14—C20	179.1 (2)
O6—C16—C15—C14	179.55 (19)	C10—N2—C14—C15	0.1 (3)
O6—C16—C15—C17	0.3 (3)	C10—N2—C11—C12	−32.2 (3)
N2—C14—C15—C17	179.81 (18)	C10—N2—C11—C13	−103.1 (3)
N2—C14—C15—C16	0.6 (3)	C10—C9—C8—O5	179.2 (2)
N2—C11—C12—C13	−110.3 (3)	C10—C9—C8—O4	−0.4 (4)
N2—C11—C13—C12	109.2 (3)	C21—N4—C24—C23	59.4 (3)
C20—C14—C15—C17	0.8 (3)	C21—N4—C19—C20	31.9 (4)
C20—C14—C15—C16	−178.49 (19)	C21—N4—C19—C18	−147.9 (2)
C20—C19—C18—C17	1.7 (4)	C15—C17—C18—C19	−1.9 (4)
C20—C19—C18—F1	−176.96 (19)	C15—C17—C18—F1	176.70 (18)
C14—N2—C10—C9	−1.4 (3)	C15—C16—C9—C10	−1.1 (3)
C14—N2—C11—C12	151.9 (2)	C15—C16—C9—C8	179.0 (2)
C14—N2—C11—C13	81.1 (3)	C7—C2—C3—C4	−1.4 (3)
C14—C20—C19—N4	−179.9 (2)	C7—C2—C1—O2	−2.5 (3)
C14—C20—C19—C18	−0.1 (3)	C7—C2—C1—O3	176.9 (2)
N4—C24—C23—N5	−53.8 (3)	C11—N2—C14—C20	−5.1 (3)
N4—C24—C23—C25	−174.7 (2)	C11—N2—C14—C15	175.90 (19)
N4—C19—C18—C17	−178.5 (2)	C11—N2—C10—C9	−177.3 (2)
N4—C19—C18—F1	2.9 (3)	C3—C2—C7—C6	2.2 (3)
N4—C21—C22—N5	55.5 (2)	C3—C2—C7—O1	−177.9 (2)
N4—C21—C22—C26	176.7 (2)	C3—C2—C1—O2	179.3 (2)
C24—N4—C19—C20	−125.9 (2)	C3—C2—C1—O3	−1.3 (4)
C24—N4—C19—C18	54.3 (3)	C6—C5—C4—C3	0.4 (4)
C24—N4—C21—C22	−59.7 (3)	N1—C5—C6—C7	178.4 (2)
C16—C9—C10—N2	1.9 (3)	N1—C5—C4—C3	−177.6 (2)
C16—C9—C8—O5	−0.9 (3)	C8—C9—C10—N2	−178.2 (2)
C16—C9—C8—O4	179.5 (2)	C23—N5—C22—C21	−54.5 (3)
N3—C17—C18—C19	177.6 (2)	C23—N5—C22—C26	−176.8 (2)
N3—C17—C18—F1	−3.8 (3)	C4—C5—C6—C7	0.4 (3)
N3—C17—C15—C14	−178.8 (2)	C1—C2—C7—C6	−176.1 (2)
N3—C17—C15—C16	0.4 (4)	C1—C2—C7—O1	3.8 (3)
C19—C20—C14—N2	179.92 (19)	C1—C2—C3—C4	176.9 (2)
C19—C20—C14—C15	−1.1 (3)	C22—N5—C23—C24	53.3 (3)
C19—N4—C24—C23	−140.8 (2)	C22—N5—C23—C25	176.3 (2)
C19—N4—C21—C22	140.8 (2)	O1—C7—C6—C5	178.3 (2)
C2—C7—C6—C5	−1.7 (3)		

### 4-[5-Amino-3-carboxy-1-cyclopropyl-6,8-difluoro-4-oxo-1,4-dihydroquinolin-7-yl]-2,6-dimethylpiperazin-1-ium 4-amino-2-hydroxybenzoate Hydrogen-bond geometry (Å, º)

*Cg*6 is the centroid of the C2–C7 ring.

**Table:**

*D*—H···*A*	*D*—H	H···*A*	*D*···*A*	*D*—H···*A*
O1—H1···O2	1.02 (4)	1.60 (4)	2.529 (3)	149 (3)
N3—H3*A*···O6	0.86	1.92	2.648 (3)	142
N3—H3*A*···O1^i^	0.86	2.52	2.965 (3)	113
N3—H3*B*···F1	0.91 (3)	2.29 (3)	2.637 (3)	102 (2)
O5—H5···O6	0.82	1.73	2.497 (3)	154
N5—H5*A*···O2^ii^	0.99 (3)	1.74 (3)	2.715 (3)	167 (2)
N5—H5*B*···O3	0.91 (3)	1.85 (3)	2.754 (3)	172 (3)
N1—H1*B*···*Cg*6^iii^	0.95 (4)	2.46 (5)	3.326 (3)	153 (4)

Symmetry codes: (i) *x*+1/2, −*y*−1/2, *z*−1/2; (ii) −*x*, −*y*, −*z*+1; (iii) −*x*+1/2, *y*−1/2, −*z*+3/2.

## References

[bb1] Bourhis, L. J., Dolomanov, O. V., Gildea, R. J., Howard, J. A. K. & Puschmann, H. (2015). *Acta Cryst.* A**71**, 59–75. 10.1107/S2053273314022207 PMC4283469 25537389

[bb2] Chauhan, P., Maitra, R., Sangwan, S., Shah, S. K. H., Chopra, S., Reddy, J. P., Nangia, A. K. & Prabhakaran, P. (2025). *Chem. Asian J*. **20**, e70356. 10.1002/asia.70356 41094723

[bb3] Desiraju, G. R. & Steiner, T. (1999). *The Weak Hydrogen Bond in Structural Chemistry and Biology*. New York: Oxford University Press Inc.

[bb4] Djaló, M., Cunha, A. E. S., Luís, J. P., Quaresma, S., Fernandes, A., André, V. & Duarte, M. T. (2021). *Cryst. Growth Des.* **21**, 995–1005.

[bb5] Dolomanov, O. V., Bourhis, L. J., Gildea, R. J., Howard, J. A. K. & Puschmann, H. (2009). *J. Appl. Cryst.* **42**, 339–341.

[bb6] Faria, A. F., de Souza, M. V. N., de Almeida, M. V. & de Oliveira, M. A. L. (2006). *Anal. Chim. Acta* **579**, 185–192. 10.1016/j.aca.2006.07.037 17723742

[bb7] Gellman, S. H. (1998). *Acc. Chem. Res.* **31**, 173–180.

[bb8] Goa, K. L., Bryson, H. M. & Markham, A. (1997). *Drugs* **53**, 700–725. 10.2165/00003495-199753040-00010 9098667

[bb9] Gunnam, A., Suresh, K., Ganduri, R. & Nangia, A. (2016). *Chem. Commun.* **52**, 12610–12613. 10.1039/c6cc06627e 27711455

[bb10] Holstein, J. J., Hübschle, C. B. & Dittrich, B. (2012). *CrystEngComm* **14**, 2520–2531.

[bb11] Jain, S., Jain, N. K. & Pitre, K. S. J. (2002). *J. Pharm. Biomed. Anal.* **29**, 795–801. 10.1016/s0731-7085(02)00178-4 12093511

[bb12] Karmakar, S., Mallik, M., Sulava, S., Modi, U., Allu, S., Sangwan, S., Tothadi, S., Prakasha Reddy, J., Vasita, R., Nangia, A. K., Alone, D. P. & Prabhakaran, P. (2025). *Biomater. Sci.* **13**, 3828–3839. 10.1039/d4bm01681e 40007480

[bb13] Kulkarni, R. M., Malladi, R. S. & Hanagadakar, M. S. (2018). *Adv. Mat. Proc*. **3**, 526–529.

[bb14] Marona, H. R. N. & Schapoval, E. E. S. (2001). *J. Pharm. Biomed. Anal.* **26**, 501–504. 10.1016/s0731-7085(01)00429-0 11489396

[bb15] McKinnon, J. J., Jayatilaka, D. & Spackman, M. A. (2007). *Chem. Commun*. pp. 3814–3816. 10.1039/b704980c 18217656

[bb16] Mehmood, T. & Reddy, J. P. (2021). In *Progress in Molecular Biology and Translational Science* vol. 185, edited by R. S. Bhosale & V. Singh, pp. 179–198. New York: Academic Press.

[bb17] Patel, K., Karmakar, S., Tothadi, S., Reddy, J. P. & Prabhakaran, P. (2024). *Chem. A Eur. J.* **30**, e202303757. 10.1002/chem.202303757 38165894

[bb18] Patel, K., Shah, S. K. H. & Prabhakaran, P. (2021). In *Progress in Molecular Biology and Translational Science* vol. 185, edited by R. S. Bhosale & V. Singh, pp. 113–136. New York: Academic Press. 10.1016/bs.pmbts.2021.06.011 34782102

[bb19] PrakashaReddy, J. & Pedireddi, V. R. (2004). *Tetrahedron* **60**, 8817–8827.

[bb20] Ramesh, V. V. E., Roy, A., Vijayadas, K. N., Kendhale, A. M., Prabhakaran, P., Gonnade, R., Puranik, V. G. & Sanjayan, G. J. (2011). *Org. Biomol. Chem.* **9**, 367–369. 10.1039/c0ob00593b 21082121

[bb21] Rigaku OD (2024). *CrysAlis PRO*. Rigaku Corporation, Yarnton, England.

[bb22] Schentag, J. J. (2000). *Clin. Ther.* **22**, 372–387. 10.1016/S0149-2918(00)89007-4 10823360

[bb23] Shah, S. K. H., Modi, U., Patel, K., James, A., N, S., De, S., Vasita, R. & Prabhakaran, P. (2023). *Biomater. Sci.* **11**, 6210–6222. 10.1039/d3bm00766a 37526301

[bb24] Shankara Prasad, H. J., Devaraju, Vinaya, Basavaraju, Y. B., Yathirajan, H. S. & Parkin, S. (2022). *Acta Cryst.* E**78**, 1257–1264. 10.1107/S2056989022008337 PMC9443794 36072519

[bb25] Sheldrick, G. M. (2015). *Acta Cryst.* C**71**, 3–8.

[bb26] Shingnapurkar, D., Butcher, R., Afrasiabi, Z., Sinn, E., Ahmed, F., Sarkar, F. & Padhye, S. (2007). *Inorg. Chem. Commun.* **10**, 459–462.

[bb27] Sowjanya, M., Sirisha, C. & Prasad, M. K. (2020). *Rese. J. Pharm. Technol.* **13**, 3587–3592.

[bb28] Spackman, M. A. & Jayatilaka, D. (2009). *CrystEngComm* **11**, 19–32.

[bb29] Spackman, P. R., Turner, M. J., McKinnon, J. J., Wolff, S. K., Grimwood, D. J., Jayatilaka, D. & Spackman, M. A. (2021). *J. Appl. Cryst.* **54**, 1006–1011. 10.1107/S1600576721002910 PMC8202033 34188619

[bb30] Vasil’ev, A. D. & Golovnev, N. N. (2014). *Russ. J. Inorg. Chem.* **59**, 322–325.

[bb31] Vasiliev, A. D. & Golovnev, N. N. (2015). *J. Struct. Chem.* **56**, 907–911.

[bb32] Wise, R. & Honeybourne, D. (1996). *J. Antimicrob. Chemother.* **37** Suppl. A, 57–63. 10.1093/jac/37.suppl_a.57 8737125

[bb33] Zhanel, G. G., Ennis, K., Vercaigne, L., Walkty, A., Gin, A. S., Embil, J., Smith, H. & Hoban, D. J. (2002). *Drugs* **62**, 13–59. 10.2165/00003495-200262010-00002 11790155

[bb34] Zhang, Y., Zhang, Y., Liu, L., Feng, Y., Wu, L., Zhang, L., Zhang, Y., Zou, D. & Liu, Y. (2022). *J. Mol. Struct.* **1250**, 131894.

